# Racial and Ethnic Factors and Opioid Use Disorder Treatment After an Emergency Department Visit

**DOI:** 10.1001/jamanetworkopen.2025.20661

**Published:** 2025-07-14

**Authors:** Edouard Coupet, Marek C. Chawarski, Kavari Hercules, Joseph L. Williams, Alexandra Murphy Ward, Patricia H. Owens, David A. Fiellin, Kathryn F. Hawk, Shara H. Martel, Gail D’Onofrio

**Affiliations:** 1 Department of Emergency Medicine, Yale School of Medicine, New Haven, Connecticut; 2 Department of Medicine, Yale School of Medicine, New Haven, Connecticut; 3 Department of Psychiatry, Yale School of Medicine, New Haven, Connecticut; 4 Department of Chronic Disease Epidemiology, Yale School of Public Health, New Haven, Connecticut; 5 Kansas City University College of Osteopathic Medicine, Kansas City, Missouri; 6 Frank H. Netter MD School of Medicine at Quinnipiac University, North Haven, Connecticut

## Abstract

**Importance:**

There are racial and ethnic disparities in opioid use disorder (OUD) treatment engagement during and after emergency department (ED) encounters.

**Objective:**

To identify patterns of barriers and facilitators to treatment engagement after an ED visit among Black, Hispanic, and White individuals with OUD.

**Design, Setting, and Participants:**

This qualitative study was conducted from June 2023 to May 2024 using in-depth semistructured individual telephone interviews. Participants with OUD were previously enrolled in a multisite study comparing 2 formulations of buprenorphine in ED patients with untreated OUD on treatment engagement.

**Exposures:**

A diagnosis of moderate-to-severe OUD and a visit to an ED.

**Main Outcomes and Measures:**

The primary outcome was identification of key themes at both the behavioral and health system levels associated with treatment engagement, identified through thematic analysis of interview data.

**Results:**

A total of 57 individuals (20 female [35.1%]; mean [SD] age, 41.7 [12.8] years; 20 Black [35.1%]; 17 Hispanic [29.8%]; 20 White [35.1%]) participated in the study. Although racial and ethnic group-specific factors existed, common barriers to treatment engagement included stigma, structural factors (eg, transportation and insurance), uncertainty navigating the health system, and mental health issues. Black participants specifically described how previous trauma and daily stress contributed to a lack of treatment engagement. Black and Hispanic participants expressed experiences of racism and mistrust within the health system. Hispanic and White participants expressed concerns about the adverse effects and taste of sublingual buprenorphine. Common facilitators included positive attitudes toward treatment and patient experiences with ED staff and stable health care access. Hispanic participants described family support as a crucial factor toward treatment engagement. Black participants expressed the importance of connecting with individuals who were abstinent.

**Conclusions and Relevance:**

In this qualitative study of 57 individuals with OUD previously treated in the ED, common themes emerged across racial and ethnic groups. However, Black, Hispanic, and White individuals with OUD encountered distinct barriers and facilitators to treatment engagement after an ED visit, such as the importance of family support among Hispanic individuals as a facilitator and experiences of racism within the health system among both Black and Hispanic individuals as a barrier. Future ED-based interventions should address disparities by reducing barriers and enhancing facilitators to improve equitable treatment access.

## Key Points

**Question:**

What are the racial and ethnic barriers and facilitators to opioid use disorder (OUD) treatment engagement after an emergency department (ED) visit?

**Findings:**

In this qualitative study of 57 participants, treatment engagement was hindered by stigma, structural barriers, health care navigation challenges, and mental health concerns across all groups; Black and Hispanic participants cited racism, and Hispanic and White participants expressed concerns about buprenorphine’s adverse effects. Key facilitators included positive attitudes to buprenorphine and ED staff, stable health care access, and social support.

**Meaning:**

Although common themes emerged, Black, Hispanic, and White individuals experienced distinct barriers and facilitators to treatment engagement after an ED visit; future work should address disparities by reducing barriers and enhancing facilitators to improve equitable treatment access.

## Teaser

This qualitative study examines patterns of barriers and facilitators to treatment engagement after an emergency department (ED) visit among Black, Hispanic, and White individuals with opioid use disorder.

## Introduction

From 2022 to 2023, the only racial and ethnic group that experienced a decrease in opioid overdose deaths was non-Hispanic White populations. During that same period, the rate of opioid overdoses remained the same in Hispanic populations and increased in Black populations.^1^ These sharp increases in deaths can be attributed to structural racism and increases in both high-potency synthetic opioids and polysubstance use.^2,3,4,5,6^

The emergency department (ED), which offers access to care for all populations 24 hours per day, 365 days a year, represents a critical opportunity to address these disparities by initiating buprenorphine and providing linkage to ongoing treatment. ED-initiated buprenorphine for untreated opioid use disorder (OUD) is an evidence-based practice that is increasingly being implemented nationwide.^7,8^ Prior qualitative studies^9,10,11^ on ED patients with OUD have not explicitly examined racial or ethnic variations. In a multisite ED study^12^ of individuals with untreated OUD, among participants seeking a referral to substance use treatment, there was a higher proportion of individuals who self-identified as American Indian or Alaska Native, Black, or other races vs White. However, Black and Hispanic individuals with OUD encounter substantial disparities in access to medications for opioid use disorder (MOUD) and subsequent treatment engagement.^5,13,14^ To our knowledge, few studies have evaluated specific barriers and facilitators to treatment engagement among both Black and Hispanic ED patients with OUD.

Project Emergency Department-Initiated Buprenorphine Validation Network Trial (ED-Innovation), the parent study, was a randomized clinical trial of 1994 ED patients with untreated OUD.^15^ In the present study, we performed a qualitative study of ED-Innovation participants after they had completed all parent study procedures. Our aim was to elicit and compare barriers and facilitators to treatment engagement among Hispanic, non-Hispanic Black (hereafter, *Black*), and non-Hispanic White (hereafter, *White*) individuals with OUD.

## Methods

### Overview of ED-Innovation

The parent study compared the effectiveness of sublingual buprenorphine vs a 7-day injectable extended-release buprenorphine across 29 ED sites on formal addiction treatment^16^ engagement at day 7. To be eligible to participate in the parent study, participants met *Diagnostic and Statistical Manual of Mental Disorders* (Fifth Edition)^17^ criteria for moderate-to-severe OUD, had a Clinical Opiate Withdrawal Scale score of 4 or greater, and a point of care urine toxicology test positive for opioids.^15^ A full description of the parent study methods has been published elsewhere.^15^

### Study Design and Participants of Qualitative Study

This qualitative study follows the Standards for Reporting Qualitative Research (SRQR) reporting guidelines.^18^ It has received approval from both the Yale University institutional review board and WCG institutional review board.

This qualitative study incorporated semistructured individual interviews by telephone of participants from 8 of the 29 ED-Innovation sites: Cooper University Health Care (Camden, New Jersey), Grady Memorial Hospital (Atlanta, Georgia), Henry Ford Hospital (Detroit, Michigan), Maine Medical Center (Portland, Maine), University of New Mexico (Albuquerque, New Mexico), University of Utah (Salt Lake City, Utah), and the Alameda Health System (Highland Hospital, Oakland, California; and San Leandro Hospital, San Leandro, California). Eligible participants self-reported their race as Black or White and ethnicity as Hispanic or non-Hispanic in the parent study, completed participation in the ED-Innovation study from July 2022 to November 2023, and agreed to be recontacted for additional research participation. We called all eligible participants and interviewed as many as responded. We obtained informed consent orally. Initially, we recruited 20 Black participants from the parent study. We then recruited 20 White and 17 Hispanic participants, matched by location, age, and sex to the Black participants for comparative racial and ethnic analyses.

### Overall Qualitative Methods

The interview guide was developed using a combination of 2 existing frameworks, The National Institute on Minority Health and Health Disparities (NIMHD) research framework^19^ and the theory of planned behavior.^20^ The NIMHD research framework is a multidomain, multilevel model that illustrates a set of relevant health determinants to understand and address health disparities.^19^ The theory of planned behavior suggests that an individual’s intention to engage in a specified behavior (ie, treatment engagement) is dictated by a combination of (1) attitudes, (2) subjective norms, and (3) perceived behavioral control toward the specified behavior, or for the purposes of this study, treatment engagement.^20^ We used this framework to elicit attitudes and subjective norms surrounding MOUD and treatment engagement, perceived behavioral control toward treatment engagement, and perceptions of the ED patient–clinician relationship.

From June 2023 to May 2024, 2 trained qualitative researchers (E.C. and K.H.) conducted 57 in-depth, semistructured telephone interviews using purposeful sampling.^21^ Both interviewers identified as Black men. The interview guide is included in the eAppendix in Supplement 1. Once developed, it was pilot tested by 1 author (E.C.) on other research team members. Interviews lasted approximately 40 minutes. Participants received a $50 gift card for participation. All interviews were audio recorded, transcribed, and deidentified using a professional transcription service. The patient enrollment flowchart is depicted in the Figure.

**Figure.**
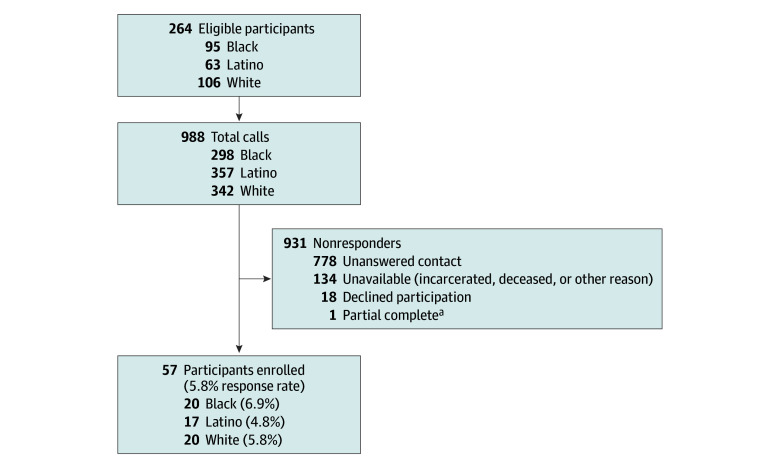
Patient Enrollment Flowchart The total number of eligible calls refers to the total number of individuals evaluated and/or calls placed to determine study participation. Nonresponders were participants who were not included in the study, with reasons such as declining to participate, unavailable, or other criteria. Participants who consented and completed all study procedures were enrolled. ^a^Participant completed a portion of the interview and finished the rest at a later time.

### Statistical Analysis

At least 2 members of the research team with training in qualitative research read and coded all transcripts independently. Transcripts were grouped and analyzed by race and ethnicity. An initial codebook was developed inductively and grounded in our theoretical framework. The research team met regularly to discuss all coded transcripts. Any disagreements were resolved by discussion until consensus was reached. The codebook was revised iteratively throughout the coding process. Once all interviews were coded, the revised codebook was reapplied to all transcripts (ie, constant comparative method^22^). The research team performed a thematic analysis within each racial and ethnic group to identify existing patterns. All interview transcripts were coded and analyzed using Dedoose software version 9.2.012 (SocioCultural Research Consultants).^23^

## Results

The sociodemographic characteristics of participants are included in Table 1. The mean (SD) age of participants was 41.7 (12.8) years; 20 participants (35.1%) were female, 20 were Black (35.1%), 17 were Hispanic (29.8%), and 20 were White (35.1%). To achieve our study aim, we concentrated on the health care system and behavioral domains of influence within the NIMHD research framework. In the health care system domain, we identified subthemes of health care stigma as a barrier and positive experiences with ED staff as a facilitator, under the theme of the ED patient–clinician relationship. In the behavioral domain, we categorized subthemes, using theory of planned behavior, into attitudes, norms, or perceived behavioral control of MOUD engagement. Within attitudes, barriers included self-stigma and concerns about buprenorphine, and a facilitator was positive attitudes toward and self-efficacy in treatment engagement. For perceived behavioral control, barriers comprised racism and mistrust within the health care system, structural factors, difficulty navigating the health care system, polysubstance use, and mental health issues, with stable health care access serving as a facilitator. Within norms, stigma from loved ones was a barrier, whereas social support was a facilitator. Table 2 includes an overview of themes and representative participant quotes. The subthemes, along with their differences and similarities across racial and ethnic groups, will be described in further detail.

**Table 1.** Sample Demographics

Characteristics	Participants, No. (%)			
	Black (n = 20)	Hispanic (n = 17)	White (n = 20)	Total (N = 57)
Age, mean (SD), y	43.0	39.0	42.7	41.7 (12.8)
Sex				
Female	5 (25.0)	7 (41.2)	8 (40.0)	20 (35.1)
Male	15 (75.0)	10 (58.8)	12 (60.0)	37 (64.9)
Health insurance, yes	14 (70.0)	15 (88.2)	17 (85.0)	46 (80.7)
Housing instability, yes^a^	5 (25.0)	6 (35.3)	9 (45.0)	20 (35.1)
Study site				
Cooper University Health Care (Camden, New Jersey)	2 (10.0)	1 (5.9)	2 (10.0)	5 (8.8)
Grady Memorial Hospital (Atlanta, Georgia)	7 (35)	1 (5.9)	3 (15.0)	11 (19.3)
Henry Ford Hospital (Detroit, Michigan)	1 (5.0)	0	1 (5.0)	2 (3.5)
Maine Medical Center (Portland, Maine)	1 (5.0)	1 (5.9)	1 (5.0)	3 (5.3)
University of New Mexico (Albuquerque, New Mexico)	0	5 (29.4)	2 (10.0)	7 (12.3)
University of Utah (Salt Lake City, Utah)	0	3 (17.6)	3 (15.0)	6 (10.5)
Highland Hospital (Oakland, California)	7 (35.0)	3 (17.6)	3 (15.0)^a^	13 (22.8)
San Leandro Hospital (San Leandro, California)	2 (10.0)	3 (17.6)	5 (25.0)	10 (17.5)

^a^ Indicates that the participant spent at least 1 night in a shelter for individuals experiencing homelessness; on the streets or in a public place; in a hotel for individuals experiencing homelessness; in someone else’s house or apartment; or in an emergency or temporary, transitional, or halfway house.

**Table 2.** Participant Quotations

Domain, barriers and facilitators, themes, and race and ethnicity	Quotation
Attitudes	
Barrier	
Self-stigma	
Black	“What would be stoppin’ me? I guess me thinkin’ that I can do it by myself. That’s the biggest part of it, thinkin’ that I don’t need any kind of … I think that’s my biggest problem if I ever wanted not to take it. Yeah, so I gotta be strong in my mind.”
Hispanic	“I think it’s okay to do it for a while, but I don’t think it’s something that you should stay on forever … then it’s like you need that to be able to function in your life, and if you’re able to get off of it then you’re able to function without anything at some point.”
White	“It’s kind of just trading one thing for another though. You’re getting put on an opiate medication after taking opiate unprescribed. There really not any benefit besides that it is more controlled, and that there’s almost no chance of you overdosing as long as you’re taking it as prescribed.”
Concerns regarding buprenorphine	
Hispanic	“I didn’t really like the Suboxone ‘cause I feel like I had relapse. Suboxone wasn’t good for me just ‘cause it didn’t help me stay clean, ‘cause when I was on Suboxone, I would just crave it … I would probably take it if they up-dose the medication.”
White	“Other than there’s definitely tooth decay and rot associated with the strips that people definitely need to know about … I found that out too late. My back teeth on the side where I put the strip, it ate those teeth away.”
Facilitator	
Positive attitudes toward and self-efficacy in treatment engagement	
Black	“Well, I get [Insurance]. Nothing I’m gonna let interfere with me and my treatment, so I don’t—I can’t really name one that I would think, that would prevent me…. I mean, out of all drugs that I’ve tried, heroin has been—always been my first love, and I don’t wanna be loved no more with heroin. It takes too much energy. Takes money, and you have to substitute eating or washing your butt, or a bar of soap. It’s not worth it at all.”
Hispanic	“No, it wouldn’t affect me ’cause, like I tell you, I have the support of my family.”
White	“I’ve been clean since that day. That emergency room visit was a life-changing event for me because the caregivers did the right thing and helped me on path where I’m totally clean. I do use Suboxone, but that is medically prescribed. It is to help me stay clean.”
Perceived behavioral control	
Barrier	
Racism and mistrust within the health care system	
Black	“Yes, because most of these treatments are ran by white people, and they do discriminate, and there’s still racism behind these walls. I’m not gonna sugarcoat that shit. Once we do get into treatment, we’re not treated fair. Like I said, we develop this mental disease as well, and can’t everybody respect that—so you get treated a way. I’ve experienced racism. I experienced discrimination just ’cause of my color. I got discriminated just how big I am. ’Cause of how big I am, my weight, how dark I am, and I’m black. I felt all three at once.”
Hispanic	“They could listen more and between some nurses and doctors, they could stop the low key racist like some things that make a lot of our cultural differences stand out, but maybe they don’t do it purposefully, I don’t know.”
Structural factors	
Black	“I kind of got thrown off, being homeless here, so I have to get right back on it, and some things I need to work on … staying positive and thinking positive, ‘cause what you think is—could be reality. Don’t gotta practice negative.”
Hispanic	“Probably just depends on where the location would be and transportation. Location and transportation would be the biggest factors.”
White	“The majority of the people that I talk to the only thing that they’re worried about is losing their [Medicaid]. Otherwise, they won’t be able to take the medication that they need to take. Other than that—that needs to be definitely addressed because my understanding is that people are losing their health benefits. How I haven’t lost mine yet? Because I’m working? I don’t know…. If that happens, a lot of people are screwed..”
Difficulty navigating the health care system	
Black	“I would need somebody, I guess, willin’ to show me which avenue to take. The study I did with you guys was only limited. It was only good for, what? Two months? A month-and-a-half? I don’t know how to get Suboxone out here like that.”
Hispanic	“I want to figure out how I can get the [buprenorphine] shot.”
White	“Yeah. I mean, making decisions and somebody contacting me or contacting somebody to find out where I can go. The who, what, where kind of thing.”
Polysubstance use	
Black	“I would just go do some marijuana or have a drink at the end of the night, but I wouldn’t take [methadone].”
Hispanic	”My family do [influence] with alcohol. That’s why I probably do alcohol…. Yeah, to my addiction too—everything.”
White	“I use meth probably like two times a day. It’s weird to explain. ’Cause when I got out of treatment, I was clean off of everything for a while, and I didn’t feel like myself. Even now that I’m not doing the fentanyl and the heroin and I’m on Suboxone, I feel good about that, but it was like something was missing out of my life. Like my puppy died.”
Mental health issues	
Black	“Living in America and being African American male, we are subject to trauma, and all of our lives we’re told that, men are not supposed to cry. Men are supposed to be tough, men are supposed to be hard, and we get a misconception that we’re not human. To have some outlet, or therapy, and to talk about the trauma that’s happened within your life, that definitely helps.”
Hispanic	“I had a death in the family, and … That hit me hard. Once I got back out here—about two days or three days after I got back out to [State], I start doin’ [Percocet]…. I started sniffin’ them. Well, for the past month and a half or so, I been doin’ every day.”
White	“What contributes to my drug use? My anxiety. I guess [opioid use] helps me in a way. There’s positives and negatives to it.”
Facilitator	
Stable health care access	
Black	“I went today and yeah, I’m still on my medicine and it’s been going okay. I’ve been doing good since I’ve been in the program. I’ve gotten a better job. I’m moving. Life has gotten a lot better.”
Hispanic	“I go to a therapist every two days. I get my Suboxone shot every three weeks. I go to the hospital weekly just to check in with the doctors. I think all those things help me stay sober.”
White	“… if I didn’t have health insurance, then I may have to pay for all this stuff out of pocket, which is expensive. It’s hard to do. First of all, everyone should get good healthcare coverage and, to my case, I have healthcare coverage. I was lucky to have that. Then, I think that we need doctors and nurses to practice radical empathy to help people with this medical problem of being addicted to drugs.”
Norms	
Barrier	
Stigma from loved ones	
Black	“I keep things to myself because I’m trying to get my life together. Just [my family] saying shit to hurt me, it just really gets to me. It makes me want to get high because I just want to ignore all that shit because it just gets to too much.”
Hispanic	“That’s one of the big reasons why I didn’t want to keep taking [buprenorphine]. It’s because [my family] would have probably looked at it as another opioid and another chance for relapse. That was one of my biggest things of not wanting to continue it as well.”
White	“If you truly want help, you cannot hide this from your friends and family. The ones that really love you, care about you will have your back no matter what. The people that don’t, they’re not your real friends/family. If you’re gonna put me down for what I’m going through, then I don’t need you. I’ve even done that with my own family. My daughter got into a fight with me over the phone once. She was staying with me, and then she [moved out].”
Facilitator	
Social support	
Black	“Yea, I have to stay in my positive circle that I built up, my support group. You can’t do it by yourself. It’s very hard. A conversation is more powerful than any type of medicine. That could be more powerful than the medicine. Just keepin’ your support group that you built up, in my sobriety. Goin’ to meetings and stuff, sittin’ in the same room with people strugglin’ with the [same] mental disease as you.”
Hispanic	“No, [not having transportation] wouldn’t affect me ’cause, like I tell you, I have the support of my family.”
White	“They would be supportive of me and have my back. I told them when I was struggling and that I needed to get help. They were there to support me, and not back away from me or to tear me down. I’m blessed.”
ED patient–clinician relationship	
Barrier	
Health care stigma	
Black	“I’m a drug addict so [ED staff] just look at me funny you know. You can feel the stigma either way. I feel like they didn’t, I don’t know, I feel like I’m a drug user, so I got, I’m also African American…. It might have something to do with each other.”
Hispanic	“How they talk to you and eyes of course, how they stare at you. ‘Cause a lot of it isn’t direct, so sometimes it’s hard to explain exactly what was going on, but you know that racism was there.”
White	“These people are sick and need help and don’t need [ED staff] looking down on them, and the doctors treating them like they’re less of a person because they’re in the ER again because of whatever addiction. This all sets up for pain somewhere.”
Facilitator	
Positive experiences with ED staff	
Black	“It didn’t make any difference in what race I was. They treated me like a human being, a person that—with respect. That’s their job to be a doctor, but they know how to separate the job from the race, your race, whatever your race is. It was all about them helping people that were on—addicted to heroin or on heroin.”
Hispanic	“They weren’t racial at all. I mean, I’m looked as a mixed race. They’re very kind to me and stuff, and they treated me the same as anyone else.”
White	“They were very respectful. There’s a lot of baggage when people talk about drug use, and this one was very clinical. I felt very safe.”

Abbreviations: ED, emergency department; ER, emergency room.

### Attitudes

#### Barriers: Self-Stigma

Overall, self-stigma, including stigmatizing attitudes toward MOUD, emerged as a barrier to treatment engagement. It came in the form of participants describing addiction as a moral dilemma. White participants feared “replacing one addiction for another” while taking MOUD, fueling their desire to limit long-term use. Like White participants, Hispanic participants expressed a desire to discontinue MOUD. Self-stigma was a reoccurring theme among Black participants who expressed that they were not engaged in treatment. Although a diagnosis of OUD was a requirement for study participation, Black participants often felt they did not need MOUD and wanted to “do it on their own.” Black participants described the need to be “mentally strong,” implying addiction is a moral dilemma, to remain abstinent.

#### Barriers: Concerns About Buprenorphine

A prominent theme among White and Hispanic participants was concerns about the adverse effects (eg, precipitated withdrawal) and taste of buprenorphine. This was not a theme among Black participants. Although White participants viewed buprenorphine as effective in treating withdrawal, they described experiencing dental issues (eg, tooth decay) from the film. White participants noted specific buprenorphine formulation preferences (ie, film vs pill) that were unable to be fulfilled. Both White and Hispanic participants had concerns about precipitated withdrawal. Hispanic participants expressed that their buprenorphine dose was subtherapeutic or supratherapeutic, which contributed to reluctance toward treatment engagement.

#### Facilitator: Positive Attitudes Toward and Self-Efficacy in Treatment Engagement

Overall, participants viewed MOUD, most notably buprenorphine, as an effective treatment for withdrawal. Participants reported more favorable beliefs toward buprenorphine than methadone, often because of stigma associated with methadone. These beliefs, including the belief that one could engage in treatment regardless of circumstances, or self-efficacy, were major facilitators toward treatment engagement. Some viewed their initial experience with ED-initiated buprenorphine as “life-changing.” Participants who perceived “few to no barriers” were confident in their ability to remain engaged in treatment. Moreover, a theme from all 3 groups noted the decision to start treatment is self-driven. Hispanic participants, more so than Black and White participants, strongly believed that family influenced their treatment engagement.

### Perceived Behavioral Control

#### Barrier: Racism and Mistrust Within the Health Care System

Overall, participants rarely expressed experiencing racism during their index ED visit. However, Black and Hispanic participants described previous experiences of racism while seeking addiction treatment within and outside of the ED. Both Black and Hispanic participants described the impact of several intersecting marginalized identities, including race, sex, socioeconomic status, and having OUD. Black and Hispanic participants cited previous incidents of discrimination that created difficulty accessing treatment. All experiences with racism were perceived to impact care, although some were more explicit than others. Black and Hispanic participants expressed health care system mistrust as a result. Racism was not a theme among White participants; however, 1 White participant stated it was harder for White individuals to access methadone.

#### Barrier: Structural Factors

Participants perceived structural factors, including housing insecurity, insurance issues, and unreliable transportation, as substantial barriers to treatment engagement. Hispanic participants cited unreliable transportation as a barrier. White participants most commonly perceived barriers as being uninsured or underinsured and having unreliable transportation. White participants also cited challenges in navigating health insurance and fear of losing it. More Black participants cited structural factors as barriers to treatment engagement than any other group. Black participants repeatedly noted unreliable transportation to make appointments and pick up medications, with some unable to afford public transportation. Black participants also identified insurance issues and housing insecurity as barriers to treatment engagement. Those facing housing insecurity expressed the mental toll of managing their situation hindered their ability to treatment engagement.

#### Barrier: Difficulty Navigating Health Care System

Participants from all groups expressed difficulty and uncertainty navigating the health care system for treatment engagement. Since many participants stated they lacked primary care clinicians, they were often unsure where to go to refill their buprenorphine. Black participants expressed a desire to continue buprenorphine but were unsure of how once they completed their initial ED prescription.

#### Barrier: Polysubstance Use

Polysubstance use was a reoccurring theme among all participants. Participants cited alcohol, cannabis, tobacco, sedative, and amphetamine-type stimulant use. Participants not engaged in treatment reported polysubstance use to mitigate withdrawal or mental health disorders. Among Hispanic participants, polysubstance use, at times, was noted to be influenced by family members with substance use.

#### Barrier: Mental Health Issues

Participants from all 3 groups noted having comorbid mental health disorders, notably depression and anxiety. Participants who continued using opioids stated that they did so to manage untreated mental health disorders (ie, self-medication). Black participants, more than any other group, described having untreated mental health disorders, previous trauma, and daily stress that contributed to ongoing opioid use and lack of treatment engagement.

#### Facilitator: Stable Health Care Access

Stable health care access was a major theme in facilitating treatment engagement among all groups. Participants described the importance of having either a primary care or addiction medicine clinician with whom to check in and prescribe buprenorphine. Participants expressed appreciation for having an ED bridge clinic to address addiction care gaps. Participants also shared the importance of having little to no out-of-pocket costs to fill their buprenorphine prescription. In some cases, participants described having MOUD delivered, alleviating transportation issues. Participants also appreciated having psychiatric services.

### Norms

#### Barrier: Stigma Among Loved Ones

Stigma from loved ones was a prominent theme among all 3 groups. Participants noted that stigma from loved ones discouraged MOUD use. Both Black and White participants shared a need to isolate from family because of stigmatization or fear of it. Both groups expressed that stigmatization and isolation contributed to a lack of treatment engagement and ongoing substance use. Despite feeling stigmatized, Hispanic participants, more than Black and White, expressed remaining connected to family, influencing treatment engagement.

#### Facilitator: Social Support

A key theme facilitating treatment engagement was the support from family and friends. Participants were reluctant to disclose their OUD diagnosis and emphasized the need for nonjudgmental support. It often came in the form of encouragement, camaraderie, and even empathy. Black participants uniquely emphasized the importance of social support from recovery groups such as Narcotics Anonymous and individuals engaged in treatment, which reinforced motivation. Hispanic participants described the support of family as a key facilitating factor to treatment engagement. Moreover, they noted that family members ensured they made appointments and even provided transportation.

### ED Patient–Clinician Relationship

#### Barrier: Health Care Stigma

Overall, participants shared they were treated well during their index ED visit. However, participants described stigma in the health care setting, particularly during previous ED visits, was a major barrier toward treatment engagement. Participants expressed that ED staff dismissed their concerns during prior encounters despite viewing the ED as their last option. Participants described instances of being prematurely discharged with unaddressed concerns. To reduce stigmatization, participants went as far as suggesting that ED patients with addiction concerns be seen in a separate ED area. Black and Hispanic participants expressed feeling that stigmatization was further exacerbated by their race and/or ethnicity. Participants from both groups described experiencing microaggressions (ie, looking at someone differently because of physical appearance) from ED staff, leading to feelings of mistrust.

#### Facilitator: Positive Experiences With ED Staff

Participants from all groups viewed their ED care as an important facilitator toward discussing their OUD and treatment engagement. Participants described their interaction with ED staff as nonjudgmental, safe, and comfortable. Participants from all groups expressed feeling that ED staff took their concerns seriously, alleviating concerns about other issues (eg, wait times or precipitated withdrawal). Black and Hispanic participants expressed feeling equal treatment during their index ED visit, and that their race or ethnicity did not diminish their care. These positive experiences established trust in ED staff.

## Discussion

In this qualitative study of Black, Hispanic, and White participants, we explored attitudes, norms, and perceived behavioral control of OUD treatment engagement and the role of the ED patient–clinician relationship in affecting treatment engagement after an ED visit. We found that Black, Hispanic, and White participants reported several shared barriers and facilitators, yet distinct factors shaped their experiences on the basis of race and ethnicity. Our findings provide further context for existing racial and ethnic disparities in ED addiction care.

Both Black and Hispanic participants expressed experiencing racism within the health care system and, as a result, intersectional stigma, or stigmatization compounded by the intersection of multiple marginalized identities (eg, race, ethnicity, and addiction).^24^ Previous studies^25,26^ have found that Black and Hispanic individuals with OUD often encounter racial discrimination within the health care system. These experiences, in addition to intersectional stigma, likely contribute to racial and ethnic inequities in OUD treatment engagement.^14,27,28,29^ Although Black participants perceived buprenorphine to be helpful in treating OUD, many expressed a preference to achieve abstinence without MOUD. This stigma may stem from the disproportionate criminalization of addiction among Black populations, perhaps reinforcing the perception of addiction as a moral failing.^30,31^ These unique experiences of racial discrimination and intersectional stigma may also explain why many Black participants perceived recovery groups as a facilitator toward treatment engagement. This finding is consistent with existing literature.^28,32,33^

Among Hispanic participants, social support from family was an important facilitator toward treatment engagement. Hispanic participants expressed receiving family support in the form of encouragement, advice, and, in some cases, transportation. Research shows that familism, a cultural value emphasizing the importance of family, increases treatment engagement among Hispanic populations with OUD.^34^ Conversely, stigma and shame from family can serve as major barriers to treatment engagement, consistent with our findings.^35,36,37^

Our findings underscore the need for holistic, culturally responsive care to address these distinct racial and ethnic factors influencing addiction care during and after ED visits. Programs with strong ties to minoritized racial and ethnic communities can increase trust and treatment engagement.^28^ ED-based interventions should be patient focused and low barrier (ie, greater flexibility) and should have strong health system and community support. ED substance use navigation, a program designed to help ED patients navigate structural barriers, is one potential solution. Anderson et al^38^ found that ED patients who received navigation care were 3 times more likely to engage in treatment compared with those who did not. Another potential low-barrier solution is ED bridge clinics, which seek to link individuals from ED to outpatient care.^39^

### Limitations

Our study has limitations. First, there is selection bias in participants’ ability and willingness to answer a telephone call and participate in a telephone interview. This may exclude individuals with disconnected telephones, limited availability, or those unable or unwilling to participate. However, to mitigate this concern, we called potential participants at different times per day of the week. Second, all study sites have experience treating individuals with OUD and as a result may have less stigma. These may limit generalizability to other settings. Third, although we matched Black, Hispanic, and White participants by sex and location to minimize confounding, some sites had higher concentrations of specific demographics (eg, more Hispanic participants at the University of New Mexico), which made matching more challenging. Geographic location may contribute to certain structural factors (eg, insurance issues in Georgia). Participants across racial and ethnic groups did not experience or report barriers and facilitators in a uniform way. Fourth, since English proficiency was required for the parent study, our findings cannot be generalized to exclusively Spanish-speaking Hispanic populations.

## Conclusions

In this qualitative study of racial and ethnic barriers and facilitators in OUD treatment engagement after an ED visit, we found that although Black, Hispanic, and White ED patients shared many similar barriers and facilitators, important differences emerged between groups. The ED is a hub for individuals with untreated OUD and has the potential to lead equity in addiction care. Future studies should evaluate culturally responsive ED-based interventions that address structural determinants of health to promote equity in addiction care.

## Notes

Supplement 1. **eAppendix.** Interview Guide

Supplement 2.
Data Sharing Statement
