# Supplementary material for: Racial and Ethnic Factors and Opioid Use Disorder Treatment After an Emergency Department Visit
Source: JAMA Netw Open. 2025 Jul 14;8(7):e2520661. doi: 10.1001/jamanetworkopen.2025.20661 (PMC12260995; doi:10.1001/jamanetworkopen.2025.20661)
Supplement: Supplement 1. — eAppendix. Interview Guide [file jamanetwopen-e2520661-s001.pdf]

Supplemental Online Content

Coupet E Jr, Chawarski MC, Hercules K, et al. Racial and ethnic factors and opioid use disorder treatment after an emergency department visit. *JAMA Netw Open.* 2025;8(7):e2520661. doi:10.1001/jamanetworkopen.2025.20661

eAppendix. Interview Guide

This supplemental material has been provided by the authors to give readers additional information about their work.

© 2025 Coupet E Jr et al. *JAMA Network Open*.

eAppendix. Interview Guide

**A. <u>Experience in the Emergency Department</u>**<br><br>**Interviewer:** *As we begin this discussion, I would like to start by talking about recent treatment in the emergency department on X/XX/XXXX.*	**Theoretical Framework**			
	**Health Care System**	**Behavioral**		
		**Attitude**	**Subjective Norm**	**Perceived Behavioral Control**
1. Tell me about your emergency department visit on X/XX/XXXX.<br>a. How do you feel you were treated by the doctors?<br>b. How do you feel you were treated by the nurses?<br>c. How do you feel you were treated by the ED staff overall?				
2. During your emergency department visit on X/XX/XXXX, what effect do you feel your race or ethnicity had on how you were treated, if any at all? If so, what was it?<br>a. Do you feel your race or ethnicity affected how you were treated by doctors/nurses/ED staff? If so, how?<br>b. Do you feel your race or ethnicity affected how seriously the ED staff took your concerns?<br>c. Do you feel your race or ethnicity affected the time it took for you to be seen?				
3. Did you discuss your drug use with a member of your emergency department care team (e.g., doctor, nurse, patient technician) during your visit? If so, can you tell me about that discussion?<br>a. What was it like to talk about your drug use with your emergency department care team?				
4. When discussing your drug use with a member of your emergency department care team, how would you feel about talking to someone with the same race or ethnicity as you?<br>a. Is it important to you that the person from your emergency department care team is a member of your race or ethnicity? If so, why? If not, why?				
5. What do you think emergency departments should be able to do to truly help you/others				

© 2025 Coupet E Jr et al. *JAMA Network Open*.

like you that may need treatment for drug use?<br>a. Do you think there is anything in particular that the emergency department can do to help you get into treatment for drug use?				
6. After an emergency department visit, how would you feel about someone contacting you to help you follow up with treatment for drug use?<br>a. What would be most helpful to you here?<br>b. What do you think would be most helpful to others like you or in your same situation?				
**B. <u>Current Experience with Substance Use</u>**<br><br>**Interviewer:** *In this part of the interview, I would like to talk about your current substance use.*	**Health Care System**	**Behavioral**		
		**Attitude**	**Subjective Norm**	**Perceived Behavioral Control**
1. Do you currently use drugs that are not prescribed for you? If so, can you please tell me about your current drug use?<br>a. Which drugs do you currently use the most? How often do you use them?<br>b. What contributes to your drug use?<br>c. Do your family/friends affect your drug use? If so, how?<br>d. How does drug use affect your day-to-day life?				
2. Do you currently drink alcohol? If so, can you please tell me about your current alcohol use?<br>a. How much do you drink? How often?<br>b. What contributes to your alcohol use?<br>c. Do your family/friends affect your alcohol use? If so, how?<br>d. How does your alcohol use affect your day-to-day life?				
3. If you are currently using drugs, what needs to happen to make you want to make a change in your drug use? If you are not currently using drugs, what needs to happen to continue not using drugs?				
4. Are you currently receiving any treatment for drug or alcohol use? If so, can you please tell me about it?<br>a. If not, what needs to happen to get you into treatment?				

© 2025 Coupet E Jr et al. *JAMA Network Open*.

5. Have you ever experienced a drug overdose? If so, can you tell me about what happened?<br>a. Were you with anyone? If so, who?<br>b. Where were you?<br>c. What, do you feel, contributed to it?				
6. What do you know about ways to prevent a drug overdose (e.g., trying small amounts before using whole amounts, having naloxone around)? Do you feel it is hard or easy to do those things? If it is hard, what makes it hard? If it is easy, what makes it easy?				
**C. <u>Perceptions on Addiction Treatment for Opioid Use Disorder</u>**<br><br>**Interviewer:** *In this part of the interview, we will be discussing your views on drug use treatment.*	**Health Care System**	**Behavioral**		
		**Attitude**	**Subjective Norm**	**Perceived Behavioral Control**
1. When someone says drug use treatment, what does that mean to you?<br>a. When you think of drug treatment, what image comes to your mind? What does it mean to you and why?				
2. How would important people in your life react if they knew you were currently receiving/seeking treatment for drug use?<br>a. How would your friends feel about you getting treatment?<br>b. How would your family feel about you getting treatment?<br>c. How would they support you?				
3. What circumstances would significantly affect your ability to receive drug treatment? And in what way?				
4. Do you feel your race or ethnicity would affect your ability to get treatment for drug use? If so, how? If not, why?				
**D. <u>Perceptions on Medications for Opioid Use Disorder</u>**<br><br>**Interviewer:** *In this final part of the interview, we will be discussing your views on medications to treat drug use disorders such as Suboxone, Subutex, buprenorphine, or methadone.*	**Health Care System**	**Behavioral**		
		**Attitude**	**Subjective Norm**	**Perceived Behavioral Control**
1. How did you feel about taking a medication to treat drug use disorders that was				

© 2025 Coupet E Jr et al. *JAMA Network Open*.

prescribed by a health professional? How did you feel about taking Suboxone either by mouth or injection during your ED visit on X/XX/XX?<br>a. Do you feel these medications are helpful to treat drug use? If so, why? If not, why?<br>b. How would/do you feel about continuing to take Suboxone that was prescribed by a health professional?<br>c. How would you feel about taking Methadone?				
2. How would important people in your life react if they knew you were given a medication by a health professional to treat your drug use disorder?<br>a. Have you talked to your friends about these medications? If you have, would they be supportive of you using them? If you have not, why not?<br>b. Have you talked to your family about these medications? If you have, would they be supportive of your using them? If you have not, why not?				
3. If you are not currently taking a medication to treat drug use disorders, what would need to happen for you to decide to start taking a medication to treat drug use disorders? If you are currently taking a medication to treat drug use disorders, what would need to happen for you to continue?				

Thank you for sharing your experiences with me today. What else do you think we should know? Thank you again.

© 2025 Coupet E Jr et al. *JAMA Network Open*.
