# Supplementary material for: Racial and Ethnic Factors and Opioid Use Disorder Treatment After an Emergency Department Visit
Source: JAMA Netw Open. 2025 Jul 14;8(7):e2520661. doi: 10.1001/jamanetworkopen.2025.20661 (PMC12260995; doi:10.1001/jamanetworkopen.2025.20661)
Supplement: Supplement 2. — Data Sharing Statement [file jamanetwopen-e2520661-s002.pdf]

Data Sharing Statement

Coupet, Jr. Racial and Ethnic Factors and Opioid Use Disorder Treatment After an Emergency Department Visit. *JAMA Netw Open*. Published July 14, 2025.
doi:10.1001/jamanetworkopen.2025.20661

Data
Data available: No
